# Genome Sequences of the Oxytetracycline Production Strain *Streptomyces rimosus* R6-500 and Two Mutants with Chromosomal Rearrangements

**DOI:** 10.1128/genomeA.00517-14

**Published:** 2014-07-17

**Authors:** Damir Baranasic, Jurica Zucko, Mridul Nair, Arnab Pain, Paul F. Long, Daslav Hranueli, John Cullum, Antonio Starcevic

**Affiliations:** a Faculty of Food Technology and Biotechnology, University of Zagreb, Zagreb, Croatia; b Computational Bioscience Research Center, Biological Environmental Sciences and Engineering Division, King Abdullah University of Science and Technology, Thuwal, Saudi Arabia; c Institute of Pharmaceutical Science, King’s College London, London, United Kingdom; d Department of Chemistry, King’s College London, London, United Kingdom; e Department of Genetics, University of Kaiserslautern, Kaiserslautern, Germany

## Abstract

The genome sequence of *Streptomyces rimosus* R6-500, an industrially improved strain which produces high titers of the important antibiotic oxytetracycline, is reported, as well as the genome sequences of two derivatives arising due to the genetic instability of the strain.

## GENOME ANNOUNCEMENT

*Streptomyces rimosus* is used industrially for the production of the important antibiotic oxytetracycline, encoded by the *otc* cluster (1). Recently a draft genome sequence of the strain *S. rimosus* ATCC 10970 was published (2). Derivatives of the strain *S. rimosus* R6 were used by the company PLIVA to produce oxytetracycline and it was believed that they were derived from a soil isolate different from ATCC 10970 (1). The strain *S. rimosus* R6-500 is an improved industrial strain which produces significant quantities of oxytetracycline. It is genetically unstable and gives rise to derivatives such as MV9, which appear to have the *otc* cluster deleted (3). However, further analyses showed that the “deleted” sequences were present at low copy numbers (estimated at about 1/1,000 per chromosome copy) and gave rise to spontaneous revertants such as MV9-R8, in which some of the “deleted” sequences regained normal copy numbers (4).

Genome sequences of the three *S. rimosus* strains R6-500, MV9, and MV9-R8 were obtained using the Illumina MiSeq sequencing platform. Paired-end reads of average single read length of 250 bp and a distance between pairs of ~400 bp were obtained. The assemblies were performed using the Velvet version 1.2.09 assembler (5) with 9 million R6-500 reads, 4 million MV9 reads, and 12 million MV9-R8 reads, using k-mers of length 51 to construct the hash tables. The assemblies yielded 243, 88, and 375 contigs, respectively, for R6-500, MV9, and MV9-R8, with *N*_50_ values of 284 kb, 179 kb, and 419 kb.

The three genome sequences were annotated using the NCBI Prokaryotic Genome Annotation Pipeline (http://www.ncbi.nlm.nih.gov/genome/annotation_prok/) (6). R6-500 had 7,387 predicted protein coding genes. The *S. rimosus* R6-500 sequences showed >99% identity with the published ATCC 10970 sequences (2), indicating that R6-500 was derived from the ATCC 10970 soil isolate. However, ~600 kb of the ATCC 10970 sequences were not present in R6-500, so the strain improvement program had resulted in extensive deletions. Compared to R6-500, the mutant MV9 had deleted ~650 kb, including the *otc* cluster, whereas MV9-R8 had regained about 190 kb of the deleted sequence. In MV9 there were rare well-aligned reads to the deleted sequences, confirming their presence at low copy numbers in the strain.

The genome sequence of *S. rimosus* R6-500 is interesting as it allows comparison of a high-producing industrial strain with the soil isolate ancestor represented by strain *S. rimosus* ATCC 10970 (2). Comparison with the strain MV9 and the revertant should throw light on this fascinating process, which allows *Streptomyces* strains to maintain large regions of the genome at low copy numbers (4).

### Nucleotide sequence accession numbers.

These whole-genome shotgun projects have been deposited at DDBJ/EMBL/GenBank under the accession numbers JJNO00000000, JMGX00000000, and JMGY00000000. The versions described in this paper are versions JJNO01000000, JMGX01000000, and JMGY01000000.
